# Comparative Effectiveness and Safety of Standard-Dose and Low-Dose Pembrolizumab in Patients with Non-Small-Cell Lung Cancer: A Multi-Institutional Cohort Study in Taiwan

**DOI:** 10.3390/cancers14051157

**Published:** 2022-02-24

**Authors:** Kai-Cheng Chang, Shih-Chieh Shao, Hui-Yu Chen, Yuk-Ying Chan, Yueh-Fu Fang

**Affiliations:** 1 Department of Pharmacy, Linkou Chang Gung Memorial Hospital, Taoyuan 333, Taiwan; thuope@cgmh.org.tw (K.-C.C.); peggy@cgmh.org.tw (H.-Y.C.); 2 Department of Pharmacy, Keelung Chang Gung Memorial Hospital, Keelung 204, Taiwan; scshao@cgmh.org.tw; 3 Department of Pharmaceutical Materials Management, Chang Gung Medical Foundation, Taoyuan 333, Taiwan; yychan@cgmh.org.tw; 4 Department of Thoracic Medicine, Chang Gung Foundation, Chang Gung Memorial Hospital, Taoyuan 333, Taiwan; 5 College of Medicine, Chang Gung University, Taoyuan 333, Taiwan

**Keywords:** non-small-cell lung cancer, pembrolizumab, weight-based dose, low-dose

## Abstract

**Simple Summary:**

The comparative effectiveness and safety of the standard dose and lower doses of pembrolizumab in non-small-cell lung cancer (NSCLC) patients still remains limited. We conducted a retrospective multi-institutional cohort study of patients newly initiating pembrolizumab in Taiwan. We found that the median overall survival (OS) and rate for all classes of immune-related adverse events (irAEs) were similar for both the standard-dose and low-dose pembrolizumab groups. Moreover, we found that patients with a pembrolizumab dose ≥1.8 mg/kg were associated with better OS than those receiving <1.8 mg/kg. Our findings suggested that a pembrolizumab dose ≥1.8 mg/kg may be the clinically minimally efficient dose.

**Abstract:**

Fixed doses at 200 mg of pembrolizumab or 2 mg/kg every 3 weeks are the standard dosages for first- and second-line treatment of non-small-cell lung cancer (NSCLC); however, in clinical practice, patients with NSCLC may receive lower doses of pembrolizumab due to drug product availability or economic factors. To date, the comparative effectiveness and safety of the standard dose and lower doses of pembrolizumab in these patients still remains limited. We conducted a retrospective cohort study by analyzing electronic medical records data from the largest multi-institutional hospital system in Taiwan. Advanced NSCLC patients newly receiving pembrolizumab with or without chemotherapy were included. Patients were classified into: (1) the standard-dose group (≥2 mg/kg), and (2) the low-dose group (<2 mg/kg). We applied inverse probability of treatment weighting (IPTW) to compare the overall survival (OS) and immune-related adverse events (irAEs) between the two treatment groups, and to evaluate the minimum clinically effective dose of pembrolizumab. We included a total of 147 NSCLC patients receiving standard-dose pembrolizumab (mean [range] age: 63.7 [58.0–73.0] years; male: 62.6%; mean [range] body weight: 60.5 [58.0–73.0] kg) and 95 patients receiving low-dose pembrolizumab (mean [range] age: 62.0 [50.0–68.8] years; male: 64.2%; mean [range] body weight: 63.9 [55.0–73.8] kg). After IPTW adjustments, the median OS was similar for both the standard-dose and low-dose pembrolizumab groups (19.3 vs. 14.3 months, log-rank *p* = 0.15). Also, the rate for all classes of irAEs was similar for both groups. We found that patients with a pembrolizumab dose ≥1.8 mg/kg were associated with better OS than those receiving <1.8 mg/kg. Our findings suggested no significant difference in OS and irAEs between patients receiving pembrolizumab ≥2 mg/kg and <2 mg/kg in clinical practice. A pembrolizumab dose ≥1.8 mg/kg may be the clinically most efficient dose.

## 1. Introduction

Lung cancer is the leading cause of cancer-related mortality and causes significant health and economic burden worldwide [1]. Non-small-cell lung cancer (NSCLC) is the most frequent (85–90%) cause of lung tumors [2]. Previously, chemotherapy and tyrosine-kinase inhibitors (TKI), based on the presence of oncogenic driver mutations, were the standard treatments for NSCLC patients [3]. However, several breakthrough novel agents have been developed for advanced NSCLC therapy which have prolonged survival for those patients. For example, antibodies of anti-program-death 1 (PD-1) or anti-program death-ligand 1 (PD-L1) (i.e., nivolumab and pembrolizumab) can disrupt the interaction between PD-1 and PD-L1, an interaction which inactivates T cells and thus permits cancer cells to evade the human immune system [4]. These novel agents have now become the first-line treatment for advanced NSCLC [3,5].

The phase 1 trial of KEYNOTE-001 designated the dosage of pembrolizumab at 2 mg/kg every 3 weeks as the standard dose for second-line treatment in NSCLC patients. However, subsequent clinical trials, including KEYNOTE-024 [6,7] and KEYNOTE-042 [8,9], chose a fixed dose of pembrolizumab 200 mg every 3 weeks as first-line treatment for NSCLC patients. Model-based study has shown that saturation in ex vivo target engagement is similar for the 2 mg/kg dose and the 200 mg fixed dose, both at 3-week intervals [10,11]. Moreover, the comparative data of pharmacokinetics, toxicity and clinical responses shows no significant differences between the weight-based dose (2 mg/kg) and the fixed-dose regimen (200 mg) of pembrolizumab [12]. The fixed-dose regimen offers greater convenience in clinical practice, but the weight-based dose regimen may offer a 24–26% cost savings for NSCLC treatment with pembrolizumab [13,14,15].

Due to the economic burden of treatment, patients may only be able to afford single 100 mg vials of pembrolizumab, the minimum strength of the drug, to treat NSCLC every 3 weeks. However, a fixed dose of 100 mg of pembrolizumab is only appropriate for patients weighing less than 50 kg, following the weight-based dosing of 2 mg/kg during each treatment course. Recent simulation data have shown that a lower dose of pembrolizumab at 1 mg/kg also provides adequate trough target engagement (with occupancy of 96.8% for patients weighing 70 kg) [10]. Freshwater, T. et al. also reported similar exposure distributions between the standard dose (≥2 mg/kg) and low dose (<2 mg/kg) of pembrolizumab [11]. To date, the evidence of treatment outcomes from different weight-based doses of pembrolizumab in NSCLC patients is still lacking; we have therefore harnessed multi-institutional electronic medical records in Taiwan to compare the effectiveness and safety between standard-dose and low-dose pembrolizumab for NSCLC in real-world clinical practice. Because the ex vivo pharmacokinetics evidence might not totally reflect the real-world pharmacodynamics evidence, our secondary objective was to explore the real-world minimum effective dose of pembrolizumab.

## 2. Materials and Methods

### 2.1. Data Sources

The Chang Gung Research Database (CGRD) contains the electronic medical records (EMR) of patients from eight Chang Gung Memorial Hospitals (CGMH) and represents about 14.0% of all cancer patients in Taiwan [16]. The CGRD contains records of individual-level demographics, health conditions, all medications records, laboratory examination results, pathological findings, radiologic results, and medical procedures. The database profiles have been described in previous literature [16]. In this study, we linked CGRD to the Taiwan Cancer Registry and the National Cause of Death Registry to obtain more details on cancer stage, cancer treatments and death outside CGMH. The data quality and validity in this dataset have been documented [17,18,19]. This study has been approved by the Institutional Review Board of the Chang Gung Medical Foundation (202001612B0C601).

### 2.2. Study Population

**Figure 1:**
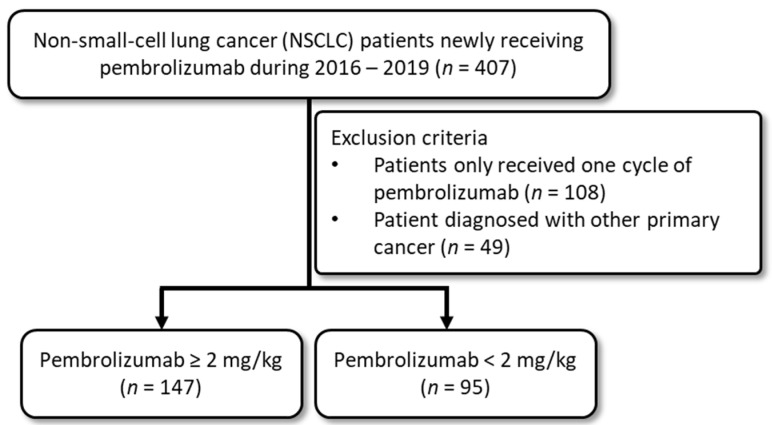
Selection of study cohort from database.

We identified all patients diagnosed with stage Ⅲ/Ⅳ NSCLC (International Classification of Diseases, Tenth version (ICD-10) disease code C34.0–C34.9) during 2015–2020 from CGRD. Patients diagnosed with other cancers were excluded because of different disease prognostic levels. We included patients newly receiving at least 2 treatment courses of pembrolizumab with or without chemotherapy between 1 January 2016 and 31 December 2019. The first date of pembrolizumab treatment was defined as the index date. The details of study cohort selection are shown in Figure 1.

### 2.3. Pembrolizumab Groups and Patients’ Demographics

Patients eligible for the study were divided into 2 groups depending on the weight-based pembrolizumab dose they received: (1) the standard-dose group (≥2 mg/kg), and (2) the low-dose group (<2 mg/kg). The patients’ weight and pembrolizumab doses for NSCLC were recorded on the index date. Pursuant to our secondary objective to explore minimum dosing, we redefined the standard group as ≥1.9 mg/kg (dosing rounded down 5%) and ≥1.8 mg/kg (dosing rounded down 10%), following a dose band strategy (10% variance) [20]. We recorded baseline information regarding patients’ characteristics such as age, Eastern Cooperative Oncology Group (ECOG) performance status, stage, histologic features, metastasis status and PD-L1 tumor proportion score on the index date. Patient comorbidities and co-medication such as alcohol behavior, smoking behavior, Charlson comorbidity index (CCI), chronic diseases and biochemical data were collected from one year prior to the index date.

### 2.4. Study Outcomes

We followed up the eligible patients from the index date to patient death, last clinical visit, loss of follow-up or 31 December 2020, whichever came first. The primary effectiveness outcome was overall survival (OS), which was defined as the time from index date to death. The secondary effectiveness outcome was the time to tumor progression (TTP), according to Response Evaluation Criteria in Solid Tumors (RECIST) version 1.1. The safety outcomes included serious adverse events post pembrolizumab treatment, defined as requiring intravenous steroid treatments for more than 3 days, and any grade immune-related adverse events (irAEs) (i.e., skin irAEs, hepatic irAEs and endocrine irAEs) according to the National Cancer Institute Common Toxicity Criteria for Adverse Events (CTCAE) version 4.0. The TTP and safety outcome assessments were judged by the multidisciplinary lung cancer conference and recorded on patients’ EMR. Detailed definitions of irAEs are shown in Supplemental Table S1.

### 2.5. Statistical Analyses

We described the patients’ characteristics by mean and interquartile range (IQR) for continuous variables, and absolute and relative frequencies for categorical variables, respectively. We compared the patients’ characteristics between the standard and low-dose pembrolizumab groups using Wilcoxon or Chi-square tests. To make the two treatment groups more homogeneous, we applied a propensity score (PS) using inverse probability of treatment weighting (IPTW) [21]. In the IPTW method, the low-dose pembrolizumab group was weighted by the inverse of the estimated PS and the standard-dose pembrolizumab group was weighted by the inverse of 1 minus the estimated PS. The Kaplan–Meier (KM) method and Cox regression model adjusted by IPTW were performed to compare the median OS and hazard ratio (HR) with 95% confidence intervals (95% CI) between the standard- and low-dose pembrolizumab groups. Moreover, we performed subgroup analyses by clinically important characteristics, including sex, age (<65 years, ≥65 years), smoking, weight (<50 kg, ≥50 kg), PD-L1 score (<50%, ≥50%), brain metastasis, concomitant chemotherapy, line of pembrolizumab therapy and experience of skin irAEs. Additionally, we also performed post hoc subgroup analyses of all included patients by line of pembrolizumab therapy, concomitant chemotherapy, experienced skin irAEs and a fixed dose of pembrolizumab (100 mg vs. 200 mg). Specifically, we reperformed the IPTW methods to generate more homogeneous comparisons between the standard- and low-dose pembrolizumab groups in each subgroup analysis. After the IPTW adjustments, we would further control the imbalanced baseline characteristics to measure the HR by using multi-variable Cox proportional hazard models. We performed all analyses using SAS Enterprise Guide, version 7.1 (SAS Institute, Cary, NC, USA). The details of SAS programs are described in Supplemental Table S2.

## 3. Results

### 3.1. Baseline Characteristics

A total of 242 NSCLC patients newly receiving pembrolizumab were included in our study. The patients’ characteristics are presented in Table 1. Of the patients, 63.2% were male with a mean age of 62.0 years (range: 56.0–72.0) and weight of 61.5 kg (52.8–69.4). More than half of the patients received pembrolizumab as first-line therapy (63.6%) for advanced NSCLC and 54.5% of the patients combined pembrolizumab with chemotherapy. Most patients had ECOG performance status less than 2 (86.8%). Among patients’ tumor histological features, 67.7% were classified as adenocarcinoma. Before pembrolizumab treatment, 114 (47.1%) had a PD-L1 tumor proportion score more than 50%.

The standard-dose group (pembrolizumab ≥2 mg/kg) consisted of 147 (60.7%) patients, and the low-dose group (pembrolizumab <2 mg/kg) consisted of the remaining 95 patients. Before the IPTW adjustment, the tumor burden, baseline characteristics and biochemical data were similar between the two groups (Table 1). However, the standard-dose group had a lower body weight (mean: 60.5 vs. 63.9 kg, *p* = 0.04) and level of alanine aminotransferase (ALT) (28.6 vs. 29.7 U/L, *p* = 0.01) than the low-dose group. Patients in the standard-dose group had a higher proportion of chronic obstructive pulmonary disease (COPD) (26.5% vs. 13.6%, *p* = 0.01) and a higher level of white blood cells (WBC) (8.4 vs. 7.6 10^3^/uL, *p* = 0.02). All baseline characteristics were comparable between the two groups after IPTW (Table 1).

### 3.2. Effectiveness Outcomes in Primary and Secondary Objectives

**Figure 2:**
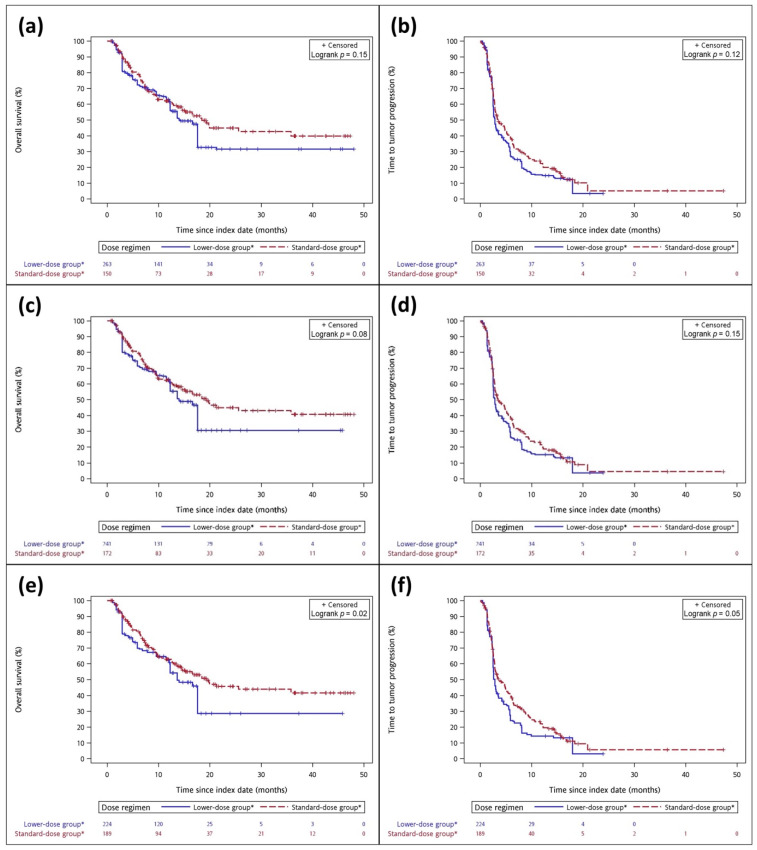
Kaplan–Meier curve of OS and TTP between standard-dose and low-dose groups after IPTW adjustment. (**a**) OS between standard-dose group and lower-dose group; (**b**) TTP between standard-dose group and lower-dose group cohort; (**c**) OS between standard-dose group (≥1.9 mg/kg) and lower-dose group; (**d**) TTP between standard-dose group (≥1.9 mg/kg) and lower-dose group; (**e**) OS between standard-dose group (≥1.8 mg/kg) and lower-dose group; (**f**) TTP between standard-dose group (≥1.8 mg/kg) and lower-dose group. Note: * The patient number was adjusted by IPTW methods.

Among these pembrolizumab new users, 107 (44.2%) patients died during the median follow-up of 10.1 months (range: 4.4–17.3 months). The median follow-up times for the standard- and low-dose groups were 9.9 and 10.6 months, respectively. After IPTW adjustment, the median OS was longer in the standard-dose group but did not reach statistical significance compared with the low-dose group (19.3 vs. 14.3 months, log-rank *p* = 0.15) (Figure 2a). The median TTP was similar in the standard- and low-dose groups (3.4 vs. 2.8 months, log-rank *p* = 0.12) (Figure 2b). For our secondary analyses, we adjusted the 2 mg/kg pembrolizumab dosing by rounding down by 5% and 10%, and we presented the baseline characteristics of redefining the standard- and low-dose groups after IPTW adjustment in Supplemental Tables S3 and S4. Taking pembrolizumab ≥1.8 mg/kg as the new standard-dose group, we found significantly better OS in the standard-dose group than in the low-dose group (HR: 0.73, 95% CI: 0.55–0.97) (Table 2). Rates for all classes of irAEs are summarized in Supplemental Table S5. The irAEs rate was similar in both groups.

### 3.3. Subgroup and Sensitivity Analyses

**Figure 3:**
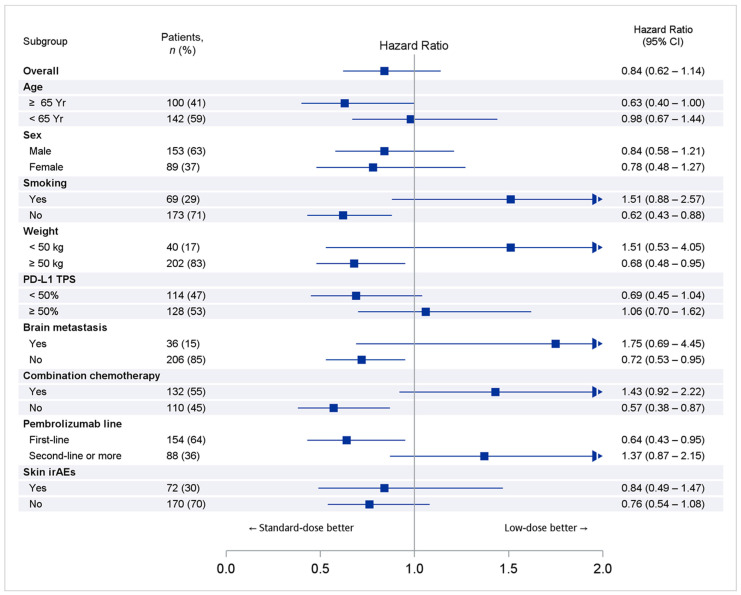
Subgroup analysis of overall survival after IPTW adjustment.

In the sensitivity analyses, we applied propensity score matching to make the two groups more comparable, and the result was consistent with the IPTW pseudo-population (HR: 0.83, 95% CI: 0.52–1.34). The primary, secondary and sensitivity analyses of OS and TTP are summarized in Table 2, Figure 2 and Supplemental Figure S1. The better OS of the standard-dose group in all subgroup analyses, while not reaching statistical significance, was similar to that of the overall population; however, statistically significant OS improvement was found in several group analyses (Figure 3), including age ≥65 years (HR: 0.63, 95% CI: 0.40–1.00), non-smokers (HR: 0.62, 95% CI: 0.43–0.88), weight ≥50 kg (HR: 0.68, 95% CI: 0.48–0.95), no brain metastasis (HR: 0.72, 95% CI: 0.53–0.98), no combination with chemotherapy (HR: 0.57, 95% CI: 0.38–0.87) and first-line pembrolizumab therapy (HR: 0.64, 95% CI: 0.43–0.95).

### 3.4. Post Hoc Subgroup Analyses of the Total Cohort

**Figure 4:**
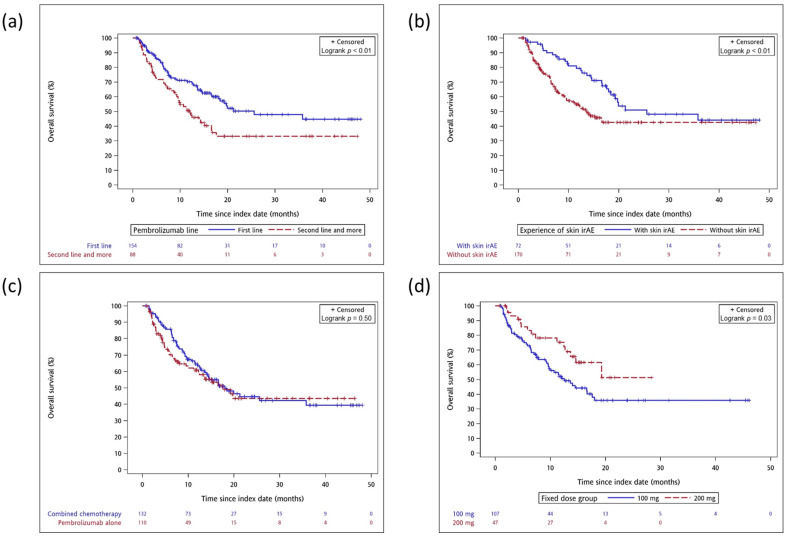
Post hoc subgroup analyses of weighted Kaplan–Meier survival curve by clinically specific group after IPTW adjustment. (**a**) Pembrolizumab first line vs. second line and more; (**b**) Skin irAEs vs. Non–skin irAEs; (**c**) Pembrolizumab alone vs. combined chemotherapy; (**d**) Pembrolizumab fixed dose 100 mg vs. 200 mg.

Post hoc subgroup analyses showed that OS was significantly better in patients receiving pembrolizumab as first-line therapy (median OS: 25.6 vs. 12.0 months; *p* < 0.01) (Figure 4a), those with skin irAEs (median OS: 25.6 vs. 13.6 months; *p* < 0.01) (Figure 4b) and those receiving fixed-dose 200 mg pembrolizumab (median OS: non–reach vs. 12.5 months; *p* < 0.05) (Figure 4d). However, the combination of chemotherapy and pembrolizumab showed similar effectiveness compared with pembrolizumab monotherapy (median OS: 18.0 vs. 17.6 months; *p* = 0.50) (Figure 4c).

## 4. Discussion

Based on Taiwan’s largest multi-institutional EMR database, our retrospective study showed no significant clinical benefits and safety difference between the standard-dose group (≥2 mg/kg) and the low-dose group (<2 mg/kg). Moreover, we found the minimum effective dose of pembrolizumab to be 1.8 mg/kg, meaning that 55.5 kg NSCLC patients could be treated with only one single 100-mg pembrolizumab vial. In our subgroup analysis, the standard dose was associated with better OS in patients aged 65 or above, non-smokers, those receiving pembrolizumab for first-line use in advanced NSCLC, those not combining with chemotherapy and those without brain metastasis.

Few real-world studies have compared clinical outcomes between standard-dose and low-dose pembrolizumab for NSCLC patients. A previous single-center study from Low, J.L. et al. in Singapore reported no significant differences in median OS between two groups receiving ≥2 mg/kg and <2 mg/kg (13.5 vs. 14.7 months) [22]. Also, pharmacokinetic studies have reported a 95% trough target engagement with dosing at 0.8 mg/kg every three weeks and saturation of PD-L1 receptors at a dose of ≥1 mg/kg [10,11,22]. Furthermore, the present study showed comparable median OS between the standard-dose and low-dose groups (19.3 vs. 14.3 months). It seems that some of the NSCLC patients who received low-dose pembrolizumab might be deriving clinical benefits at the low dose.

Rate of irAEs in our study were slightly higher than those reported in the pivotal phase 3 studies [6,7,23,24] and similar to those reported in the previous real-world study [25]. More frequent contact and intensive education, and highly selected patients in the well-controlled trial setting may contribute this discrepancy. For example, poorer performance status in our and previous real-world study population than previous trials’ population might have a higher incidence of irAEs. Nevertheless, rate of irAEs were not significantly different between the standard-dose and low-dose groups and we suggested that healthcare providers should monitor the occurrence of irAEs regardless of dosage.

The Low. J.L. et al. study also showed better median OS but no significant difference between fixed-dose 200 mg and 100 mg (19.8 vs. 14.3 months) [22]. However, our study showed that a fixed-dose 200 mg of pembrolizumab was associated with significantly better OS than fixed-dose 100 mg (non-reach vs. 12.5 months). Previous systematic reviews showed that PD-L1 expression was associated with better OS for NSCLC patients under pembrolizumab treatment [26,27]. Hence, comparing patient data of our study with those of Low, J.L. et al., we found that 68% and 39%, respectively, of the fixed-dose 200 mg patients had PD-L1 expression ≥50%, and 43% and 68%, respectively, of the fixed-dose 100 mg patients had PD-L1 expression ≥50%. Although the percentage of PD-L1 expression ≥50% between fixed-dose 200 mg and 100 mg did not reach the statistical significance (68% vs. 43%, *p* = 0.06) in our study, it might be an important factor that contributed different median OS. Otherwise, evidence directly comparing pembrolizumab alone versus pembrolizumab combined with chemotherapy for NSCLC patients remains scant. Previous indirect comparison evidence has shown controversial outcomes in OS and improved outcomes in progression-free survival (PFS) [28,29,30]. Again comparing our study with Low, J.L. et al., 77% and 35% of patients, respectively, received fixed-dose 200 mg pembrolizumab alone, while 40% and 74%, respectively, received fixed-dose 100 mg pembrolizumab alone. Details of baseline characteristics of our study and the Low, J.L. et al. study between fixed-dose 200 mg and the 100 mg group are summarized in Supplemental Table S6. Differences in demographics (i.e., PD-L1 expression) and treatment patterns between countries may give rise to the observed differences in OS between the two groups (200 mg and 100 mg fixed-dose groups).

To our knowledge, there has been no analysis based on real-world evidence to determine the minimum clinically effective dose of pembrolizumab for NSCLC treatment. A model-based study from the KEYNOTE–001 trial showed saturation of ex vivo target engagement in blood beginning at a dose of ≥1 mg/kg every 3 weeks [10]. Our study tried to determine the lowest cutoff value based on real-world clinical practice, and we found that overall survival differed significantly between the weight-based dose ≥1.8 mg/kg and <1.8 mg/kg. Asian real-world evidence showed that the median pembrolizumab dose was 1.85 mg/kg (range: 1.24–3.2 mg/kg every 3 weeks), with their patients receiving a fixed dose at 100 mg^2^. Due to the small sample size, they did not analyze clinical outcomes for the lowest-dose cut-off value. Our study provides evidence that the ex vivo pharmacokinetics models may not fully apply to real-world clinical practice. However, due to the limitations of our retrospective study design, pharmacokinetics data (i.e., blood concentration of pembrolizumab) was not available to allow a comparison with previous model-based studies [10,11].

In our subgroup analyses, standard-dose groups were associated with significantly better OS in some predefined subcohorts. In previous randomized controlled trials, the clinical benefits of immune checkpoint inhibitors (ICIs) were not significantly better in patients aged 65 or over, and nonsmokers, as compared to placebo or chemotherapy. Recent meta-analyses, pooling all published randomized trials of standard-dose ICIs, showed consistent clinical benefits among elderly and non-elderly patients [31]. However, ICIs might be less effective in nonsmokers at the standard dose, and lower dosing of ICIs might worsen clinical outcomes. Moreover, patients receiving pembrolizumab treatment alone and those receiving it as first-line therapy had better OS at the standard dose. These findings correspond to the pivotal phase 3 study and current treatment guidelines [3,6,7]. Taking together our findings and previous literature, we suggest that patients aged ≥65 and nonsmoking patients should receive the standard dose of pembrolizumab for the treatment of NSCLC.

Our study provides real-world evidence from Taiwan on different doses of pembrolizumab therapy for advanced NSCLC. However, several limitations should be noted before interpreting the results. First, due to the inherent nature of retrospective designs, possible impacts from confounding factors must be acknowledged. To mitigate this, we applied IPTW and PS matching methods to make the comparisons more homogeneous. Second, patients may become lost to follow-up in the study hospitals. Therefore, we linked our EMR database to the national death records database to capture any deaths outside the study hospitals, as a result of which we consider our estimates of median OS unbiased [32]. Third, incidence of irAEs might be underestimated due to some grade 1 adverse events only recorded in unstructured medical records. NSCLC patients receiving pembrolizumab with irAEs had better clinical outcomes compared to those without irAEs [25,33]. However, we defined irAEs using drug records and laboratory examinations and found that the incidence of irAEs was similar for the two groups. We therefore consider that incidence of irAEs is unlikely to have been underestimated.

## 5. Conclusions

This multi-institutional cohort study suggested no significant differences in OS and irAEs between NSCLC patients receiving pembrolizumab ≥2 mg/kg (standard-dose group) and <2 mg/kg (low-dose group) in Taiwan. NSCLC patients with pembrolizumab dose ≥1.8 mg/kg were associated with better OS than those receiving <1.8 mg/kg. Future studies on the effectiveness and safety from the lower dose of pembrolizumab are suggested to replicate our findings.

## Figures and Tables

**Table 1:** Baseline characteristics.

Characteristic	Total Patients (*n* = 242)	Original Cohort			IPTW Cohort *		
		Standard-Dose (*n* = 147)	Low-Dose (*n* = 95)	*p* Value	Standard-Dose (*n* = 263)	Low-Dose (*n* = 150)	*p* Value
Age, mean years (range)	62.0 (56.0–72.0)	63.7 (58.0–73.0)	62.0 (54.0–72.0)	0.09	63.6 (58.0–73.0)	63.7 (58.0–73.0)	0.92
Male (%)	63.2%	62.6%	64.2%	0.79	61.5%	67.5%	0.17
Body weight, mean kg (range)	61.5 (52.8–69.4)	60.5 (50.0–68.8)	63.9 (55.0–73.8)	0.03	60.4 (50.0–68.8)	65.4 (59.5–73.8)	<0.01
Alcohol (%)	17.4%	15.6%	20.0%	0.38	16.5%	14.0%	0.30
Smoking (%)	28.5%	29.9%	26.3%	0.50	29.5%	29.8%	0.95
CCI, mean (range)	5.4 (4.0–6.0)	5.3 (4.0–6.0)	5.0 (4.0–6.0)	0.37	5.5 (4.0–6.0)	5.5 (5.0–6.0)	0.94
Line of pembrolizumab (%)							
1	63.6%	64.6%	62.1%	0.53	64.2%	58.4%	0.20
≥2	36.4%	35.4%	37.9%		35.8%	41.6%	
Concomitant chemotherapy (%)	54.5%	50.3%	61.0%	0.10	54.0%	51.1%	0.52
ECOG (%)							
<2	86.8%	84.4%	90.5%	0.16	85.1%	88.8%	0.23
≥2	13.2%	15.6%	9.5%		14.9%	11.2%	
Stage (%)							
3	13.2%	12.2%	14.7%	0.57	13.0%	12.5%	0.86
4	86.8%	87.8%	85.3%		87.0%	87.5%	
Histologic features (%)							
Adenocarcinoma	67.7%	66.7%	69.5%	0.76	67.7%	76.1%	0.08
Squamous	24.4%	24.5%	24.2%		23.7%	18.9%	
Others	7.9%	8.8%	6.3%		8.6%	5.0%	
Metastasis status (%)							
Brain	14.9%	14.9%	14.7%	0.96	14.6%	16.5%	0.59
Bone	31.0%	34.6%	25.2%	0.12	33.6%	33.6%	0.99
Liver	12.0%	10.8%	13.6%	0.51	12.3%	9.8%	0.36
Others	84.7%	85.0%	84.2%	0.86	84.9%	87.2%	0.46
PD-L1 tumor proportion score (%)							
<1%	11.6%	11.7%	11.6%	0.50	12.1%	9.5%	0.86
1–49%	21.9%	20.4%	24.2%		22.4%	21.3%	
≥50%	47.1%	51.0%	41.0%		48.0%	51.2%	
Unknown	19.4%	16.9%	23.2%		17.5%	18.0%	
Comorbidity							
Hypertension (%)	28.5%	30.6%	25.3%	0.36	29.0%	35.7%	0.12
Diabetes (%)	14.8%	15.6%	13.6%	0.67	14.5%	15.7%	0.71
Dyslipidemia (%)	11.9%	12.2%	11.5%	0.87	13.1%	8.4%	0.08
Ischemic heart disease (%)	5.3%	4.1%	7.3%	0.26	4.4%	3.2%	0.49
Heart failure (%)	1.6%	0.6%	3.1%	0.13	0.5%	0.9%	0.57
Cerebrovascular disease (%)	4.5%	4.7%	4.2%	0.84	5.2%	2.5%	0.11
Hypothyroidism (%)	0.4%	0.6%	0.0%	0.42	0.5%	0.0%	0.20
COPD (%)	21.4%	26.5%	13.6%	0.01	23.4%	22.1%	0.75
Chronic kidney disease (%)	7.0%	8.8%	4.2%	0.16	8.8%	11.7%	0.29
Biochemical data							
eGFR (mL/min/1.73 m^2^)	98.7 (71.6–118.2)	100.7 (69.8–119.1)	95.6 (77.4–117.8)	0.35	100.8 (69.8–119.1)	90.2 (77.4–117.8)	0.06
ALT (U/L)	29.0(14–37)	28.6 (13.0–36.0)	29.7 (18.0–39.0)	0.01	28.5 (13.0–36.0)	29.4 (18.0–39.0)	0.68
AST (U/L)	31.4(19–33)	31.6 (18.0–31.0)	31.0 (20.0–36.0)	0.05	31.6 (18.0–31.0)	28.3 (20.0–36.0)	0.22
Total bilirubin (mg/dL)	0.6 (0.4–0.6)	0.7 (0.4–0.6)	0.6 (0.4–0.7)	0.32	0.7 (0.4–0.6)	0.7 (0.4–0.7)	0.92
Fasting glucose (mg/dL)	126.0 (97–132)	126.3 (98–133)	125.6 (94–131)	0.07	126.1 (98–133)	118.8 (94–131)	0.13
HbA1c (%)	6.2 (5.6–6.5)	6.2 (5.6–6.5)	6.3 (5.6–6.7)	0.29	6.2 (5.6–6.5)	6.3 (5.6–6.7)	0.44
WBC (10^3^/uL)	8.8 (5.9–10.9)	9.4 (6.1–11.2)	8.0 (5.7–9.8)	0.02	9.4 (6.1–11.2)	8.6 (5.7–9.8)	0.06
Hemoglobin (g/dL)	11.6 (10.5–13.0)	11.5 (10.4–13.1)	11.7 (10.6–13.0)	0.31	11.5 (10.4–13.1)	11.6 (10.6–13.0)	0.65
Platelet (10^3^/uL)	273.7 (187.5–328.5)	277.8 (196–323)	267.3 (177–344)	0.27	278.7 (196–323)	269.0 (177–344)	0.61

Abbreviations—CCI: Charlson comorbidity index, ECOG: Eastern Cooperative Oncology Group, PD-L1: programmed death-ligand 1, COPD: chronic obstructive pulmonary disease, eGFR: estimated glomerular filtration rate, ALT: alanine aminotransferase, AST: aspartate aminotransferase, TSH: thyroid-stimulating hormone, WBC: white blood cell. Note: Continuous variables are expressed as mean (Q1–Q3) and dichotomous variables are expressed as percentage (%). * Since the patients were adjusted by IPTW, the total patient number was not identical to that in the original cohort.

**Table 2:** Sensitivity analyses of overall survival and time to tumor progression between standard-dose and low-dose groups after IPTW adjustment.

Sensitivity Analyses	Overall Survival (HR, 95% CI)	Time to Tumor Progression (HR, 95% CI)
Pembrolizumab ≥ 2 mg/kg as standard group ^†^	0.84 (0.62–1.14)	0.85 (0.68–1.05)
Pembrolizumab ≥ 1.9 mg/kg as standard group ^†^	0.78 (0.58–1.03)	0.85 (0.69–1.06)
Pembrolizumab ≥ 1.8 mg/kg as standard group ^†^	0.73 (0.55–0.97) *	0.81 (0.66–1.01)
Propensity score matching cohort (pembrolizumab ≥2 mg/kg as standard group)	0.83 (0.52–1.34)	0.86 (0.61–1.20)

Abbreviations—HR: hazard ratio, CI: confidence interval: Note: ^†^ adjusted by inverse probability of treatment weighting; * *p* < 0.05; Propensity score model covariates include: age, sex, Eastern Cooperative Oncology Group, alcohol habit, smoking habit, line of pembrolizumab, concomitant chemotherapy, Charlson comorbidity index, histologic features, metastasis status, programmed death-ligand 1 tumor proportion score and chronic diseases.

## Data Availability

The data presented in this study are available on request from the corresponding author.

## Supplementary Materials

The following supporting information can be downloaded at: https://www.mdpi.com/article/10.3390/cancers14051157/s1, Figure S1: Kaplan-Meier curve of OS and TTP between standard-dose and low-dose groups before IPTW adjustment; Table S1: Definition of immune-related adverse events, Table S2: SAS programs for the data analyses in this study, Table S3: Baseline characteristics (re-defined the standard group as ≥1.9 mg/kg), Table S4: Baseline characteristics (re-defined the standard group as ≥1.8 mg/kg), Table S5: Summary of immune-related adverse events, Table S6: Baseline characteristics of our study and Low J.L. et al. study between fixed-dose 200 mg and 100 mg group.
