# Supplementary material for: Comparative Effectiveness and Safety of Standard-Dose and Low-Dose Pembrolizumab in Patients with Non-Small-Cell Lung Cancer: A Multi-Institutional Cohort Study in Taiwan
Source: Cancers (Basel). 2022 Feb 24;14(5):1157. doi: 10.3390/cancers14051157 (PMC8909459; doi:10.3390/cancers14051157)
Supplement: Supplementary file 1 [file cancers-14-01157-s001.zip › cancers-1572611-supplementary.pdf]

Supplementary Materials: Comparative Effectiveness and Safety of Standard-Dose and Low-Dose Pembrolizumab in Patients with Non-Small-Cell Lung Cancer: A Multi-Institutional Cohort Study in Taiwan

Kai-Cheng Chang, Shih-Chieh Shao, Hui-Yu Chen, Yuk-Ying Chan and Yueh-Fu Fang

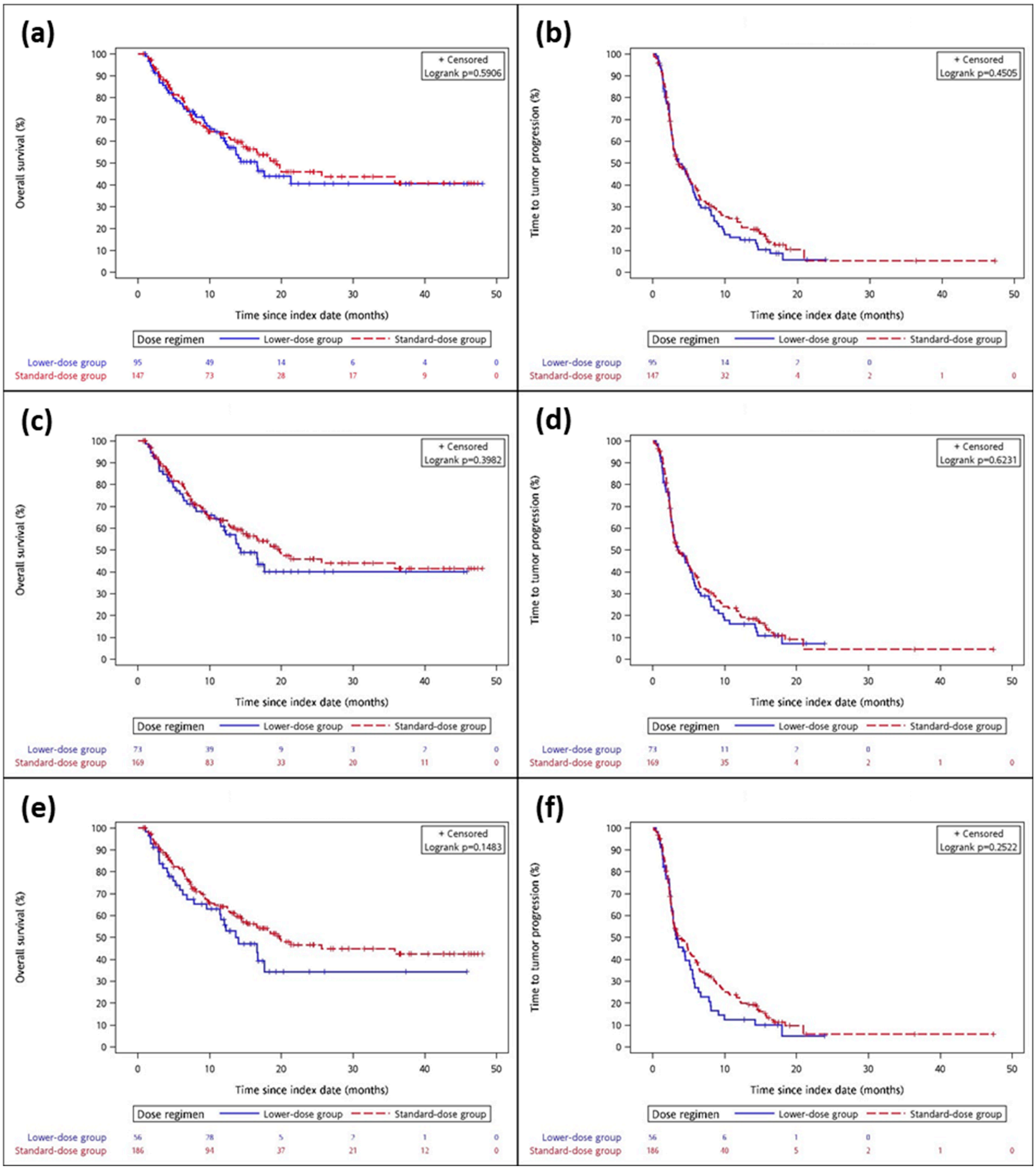

Figure S1. Kaplan-Meier curve of OS and TTP between standard-dose and low-dose groups **before** IPTW adjustment. (**a**) OS between standard-dose group (≥2 mg/kg) and lower-dose group (**b**) TTP between standard-dose group (≥2 mg/kg) and lower-dose group (**c**) OS between standard-dose group (≥1.9 mg/kg) and lower-dose group (**d**) TTP between standard-dose group (≥1.9 mg/kg) and lower-dose group (**e**) OS between standard-dose group (≥1.8 mg/kg) and lower-dose group (**f**) TTP

between standard-dose group (≥1.8 mg/kg) and lower-dose group. Note: *IPTW pseudo-population number.

Table S1. Definition of immune-related adverse events.

IrAEs Category	Definition
Skin irAEs	● Topic steroid uses and/or oral anti-histamine agents records after index date
Hepatic irAEs	● AST or ALT > ULN and/or total bilirubin > ULN
Diabetic irAEs	● Fasting glucose value > ULN
Thyroid irAEs	● Hypothyroidism: Elevated TSH, normal or low FT4.<br>● Hyperthyroidism: Suppressed TSH and high-normal or elevated FT4 and/or triiodothyronine.

Abbreviations - irAEs: immune-related adverse effects, AST: aspartate aminotransferase, ALT: alanine aminotransferase, ULN: upper limit of normal, TSH: thyroid-stimulating hormone, FT4: free thyroxine.

Table S2. SAS programs for the data analyses in this study.

```
/************************************************************************************
[a.cohort_mainfile] is analytical main file which has
• De-identical ID (ID)
• Pembrolizumab start date (index_date)
• Censor date (Censor_date)
• Standard- and low- dose group (Pembro2_YN)
• Sex (sex)
• Age (age)
• smoking (smoking_YN)
• Alcohol (Allochol_YN)
• ECOG >= 2 or < 2 (ECOG2_YN)
• PD-L1 >= 50% or not (PDL150_YN)
• Histologic (Histologic_Adeno_YN)
• stage 3 or 4 (stage)
• Bone metastasis (Bone_YN)
• Brain metastasis (Brain_YN)
• Liver metastasis (Liver_YN)
• Other organ metastasis (Other_YN)
• Combined chemotherapy (Combination_CM_YN)
• Line of pembrolizumab (Pembro_2line_YN)
• Charlson comorbidity index (CCI_total)
• Comorbidity (DM HTN Dyslipidemia IHD HF Cerebro COPD CKD Hypothyroidism)
• Lab data (eGFR ALT AST Total_bili sugar HbA1c WBC Hb PLT)
[a.pembro_mainfile_ps] is analytical main file which has
• Standard- and low- dose group (Pembro2_YN)
• IPTW score (_ATE_)
•Time from index to event date or censor date (Event_months)
•Event or not (Event)
************************************************************************************/


ODS graphics ON;
/******Propensity score calculation*******/
/**************Adjusted KM curve*********************************/
/*************Reference
Yuan, Yang, Yiu-Fai Yung, and Maura Stokes. "Propensity score methods for causal inference with the PSMATCH procedure."
Proceedings at the SAS Global Forum 2017 Conference. Cary, NC. 2017.
                                                          *****************************************/
```

```
proc PSMATCH data=a.cohort_mainfile region=allobs;
class Pembro2_YN
SEX Smoking_YN Alochol_YN   ECOG2_YN PDL150_YN Histologic_Adeno_YN stage
Bone_YN Brain_yn Liver_yn Other_yn
Combination_CM_yn Pembro_2line_yn
DM HTN Dyslipidemia IHD HF Cerebro   COPD CKD Hypothyroidism
;
psmodel Pembro2_YN (treated="1")=
SEX Smoking_YN Alochol_YN ECOG2_YN PDL150_YN Histologic_Adeno_YN stage
Bone_YN Brain_yn Liver_yn Other_yn
Combination_CM_yn Pembro_2line_yn
DM HTN Dyslipidemia IHD HF Cerebro COPD CKD Hypothyroidism
Age CCI_total
eGFR ALT AST Total_bili sugar HbA1c WBC Hb PLT
;
assess PS var=(
SEX Smoking_YN Alochol_YN   ECOG2_YN PDL150_YN Histologic_Adeno_YN stage
Bone_YN Brain_yn Liver_yn Other_yn
Combination_CM_yn Pembro_2line_yn
DM HTN Dyslipidemia IHD HF Cerebro   COPD CKD Hypothyroidism
Age CCI_total
eGFR ALT AST Total_bili sugar HbA1c WBC Hb PLT
)/varinfo plots=all weight=ATEWGT;
output out(obs=all)=a.pembro_mainfile_ps ATEWGT=_ATE_;
run;



/*******size of the pseudo population********/


proc sql;
select sum(_ATE_) as Pseudo
from a.pembro_mainfile_ps;
quit;
```

```
/**************Adjusted KM curve**********************************/
/*************Reference
Xie J, Liu C. "Adjusted Kaplan-Meier estimator and log-rank test with inverse probability of treatment weighting for survival data."
Published correction appears in Stat Med. 2007 May 10;26(10):2276.
                                                                  *****************************************/
proc lifetest data=a.pembro_mainfile_ps plots=survival(test atrisk(outside maxlen=30));
time    Event_months*Event(0);
strata Pembro2_YN;
freq _ATE_;
run;
ODS GRAPHICS OFF;

/******Adjust Cox regression model**********************************/
  /*************Reference
Austin, Peter C. "The use of propensity score methods with survival or time-to-event outcomes: reporting measures of effect similar to those used in randomized experiments."
Published at Statistics in medicine vol. 33,7 (2014): 1242-58. doi:10.1002/sim.5984
                                                                  *****************************************/

proc phreg data=a.pembro_mainfile_ps;
  class Pembro2_YN (ref='0');
  model Event_months*Event(0)=Pembro2_YN/risklimits;
  freq _ATE_;
  run;
```

Table S3. Baseline characteristics (re-defined the standard group as ≥ 1.9 mg/kg).

Characteristics	Total Patients (*n* = 242)	Re–Defined Cohort Standard–Dose (*n* = 169)	Re–Defined Cohort Low–Dose (*n* = 73)	Re–Defined Cohort *p* Value	IPTW cohort* Standard–Dose (*n* =172)	IPTW cohort* Low–Dose (*n* = 214)	IPTW cohort* *p* Value
Age, mean years (range)	62.0 (56.0–72.0)	63.9 (57.0–73.0)	61.2 (55.0–69.0)	0.09	63.1 (56.0–73.0)	63.2 (58.0–74.0)	0.94
Male (%)	63.2%	63.9%	61.6%	0.73	62.8%	67.1%	0.32
Body weight, mean kg (range)	61.5 (52.8 – 69.4)	61.1 (50.5–69.5)	63.6 (56.4–67.8)	0.13	60.7 (50.0–69.5)	65.4 (59.6–73.8)	< 0.01
Alcohol (%)	17.4%	16.5%	19.1%	0.62	17.4%	13.0%	0.17
Smoking (%)	28.5%	29.5%	26.0%	0.57	29.3%	30.1%	0.84
CCI, mean (range)	5.4 (4.0 – 6.0)	5.4 (4.0–6.0)	5.3 (5.0–6.0)	0.56	5.4 (4.0–6.0)	5.4 (5.0–6.0)	0.93
Line of pembrolizumab (%)							
1	63.6%	63.9%	63.0%	0.89	63.6%	58.3%	0.22
≥2	36.4%	36.1%	37.0%		36.4%	41.7%	
Concomitant chemotherapy (%)	54.5%	50.3%	64.3%	0.04	54.2%	50.6%	0.43
ECOG (%)							
<2	86.8%	85.2%	90.4%	0.27	85.9%	88.5%	0.39
≥2	13.2%	14.8%	9.6%		14.1%	11.5%	
Stage (%)							
3	13.2%	10.7%	19.2%	0.07	11.4%	13.7%	0.44
4	86.8%	89.3%	80.8%		88.6%	86.3%	
Histologic features (%)							
Adenocarcinoma	67.7%	68.0%	67.1%	0.98	68.8%	76.0%	0.19
Squamous	24.4%	24.3%	24.7%		23.7%	18.5%	
Others	7.9%	7.7%	8.2%		7.5%	5.4%	
Metastasis status (%)							
Brain	14.9%	15.9%	12.3%	0.46	15.6%	15.8%	0.93
Bone	31.0%	33.7%	24.7%	0.16	33.1%	34.1%	0.79
Liver	12.0%	11.8%	12.3%	0.91	13.1%	8.9%	0.13
Others	84.7%	86.4%	80.8%	0.26	86.3%	86.3%	0.99
PD–L1 tumor proportion score (%)							
<1%	11.6%	11.2%	12.3%	0.34	11.7%	9.5%	0.60
1–49%	21.9%	19.9%	28.7%		20.7%	22.5%	
≥ 50%	47.1%	50.9%	38.4%		47.9%	51.6%	
Unknown	19.4%	18.0%	20.6%		19.7%	16.4%	
Comorbidity							
Hypertension (%)	28.5%	30.7%	23.3%	0.23	28.9%	36.4%	0.07
Diabetes (%)	14.8%	15.9%	12.3%	0.46	14.7%	15.7%	0.75
Dyslipidemia (%)	11.9%	11.8%	12.3%	0.91	12.4%	8.5%	0.14

Ischemic heart disease (%)	5.3%	5.3%	5.5%	0.96	5.2%	2.4%	0.09
Heart failure (%)	1.6%	2.3%	0%	0.18	1.84%	0.0%	0.07
Cerebrovascular disease (%)	4.5%	4.1%	5.4%	0.64	4.5%	2.8%	0.27
Hypothyroidism (%)	0.4%	0.6%	0.0%	0.51	0.4%	0.0%	0.25
COPD (%)	21.4%	25.4%	12.3%	0.02	22.4%	22.8%	0.90
Chronic kidney disease (%)	7.0%	8.2%	4.1%	0.24	8.1%	12.5%	0.11
Biochemical data							
eGFR (mL/min/1.73 m^2)	98.7 (71.6–118.2)	98.8 (57.0–73.0)	98.4 (77.5–120.0)	0.95	100.7 (71.2–118.2)	87.2 (67.9–111.7)	0.06
ALT (U/L)	29.0(14–37)	28.5 (13.0–36.0)	30.3 (18.0–39.0)	0.62	28.7 (14.0–36.0)	30.1 (18.0–41.0)	0.63
AST (U/L)	31.4(19–33)	31.9 (18.0–33.0)	30.2 (20.0–34.0)	0.72	32.0 (19.0–33.0)	28.4 (19.0–36.0)	0.29
Total bilirubin (mg/dL)	0.6 (0.4–0.6)	0.6 (0.4–0.6)	0.5 (0.4–0.7)	0.62	0.6 (0.4–0.6)	0.6 (0.4–0.7)	0.94
Fasting glucose (mg/dL)	126.0(97–132)	126.6 (97.0–134.0)	124.5 (95.0–129.0)	0.82	124.5 (97.0–132.0)	118.0 (99.0–121.0)	0.34
HbA1c (%)	6.2 (5.6–6.5)	6.2 (5.6–6.5)	6.3 (5.6–6.6)	0.80	6.2 (5.6–6.5)	6.2 (0.4–0.7)	0.88
WBC (10^3/uL)	8.8 (5.9–10.9)	9.1 (5.9–11.1)	8.1 (5.9–9.9)	0.06	9.1 (5.8–11.1)	8.5 (6.4–10.2)	0.29
Hemoglobin (g/dL)	11.6 (10.5–13.0)	11.6 (10.4–13.2)	11.7 (10.6–12.9)	0.73	11.6 (10.5–13.2)	11.6 (10.6–12.6)	0.76
Platelet (10^3/uL)	273.7 (187.5–328.5)	273.1 (189.0–325.5)	274.9 (183.5–344.0)	0.91	271.3 (186.0–323.0)	269.9 (191.0–344.0)	0.92

Abbreviations - CCI: Charlson comorbidity index, ECOG: Eastern Cooperative Oncology Group, PD-L1: programmed death ligand 1, COPD: chronic obstructive pulmonary disease, eGFR: estimated glomerular filtration rate, ALT: alanine aminotransferase, AST: aspartate aminotransferase, TSH: thyroid-stimulating hormone, WBC: white blood cell. Note: Continuous variables are expressed as mean (Q1-Q3) and dichotomous variables are expressed as percentage (%) *Since the patients were adjusted by IPTW, the total patient number was not identical to that in the original cohort.

Table S4. Baseline characteristics (re-defined the standard group as ≥ 1.8 mg/kg).

**Characteristics**	**Total Patients (*n* = 242)**	**Original Cohort**			**IPTW Cohort***		
		**Standard–Dose (*n* = 186)**	**Low–Dose (*n* = 56)**	***p* Value**	**Standard–Dose (*n* = 189)**	**Low–Dose (*n* = 224)**	***p* Value**
Age, mean years (range)	62.0 (56.0–72.0)	63.8 (57.0–73.0)	60.6 (54.0–67.0)	0.06	63.1 (56.7–73.0)	63.2 (58.0–74.0)	0.94
Male (%)	63.2%	64.5%	58.9%	0.44	62.8%	67.1%	0.32
Body weight, mean kg (range)	61.5 (52.8–69.4)	61.3 (51.0–70.0)	63.7 (59.2–67.3)	0.20	60.7 (50.0–69.5)	65.4 (59.6–73.8)	<0.01
Alcohol (%)	17.4%	16.1%	21.4%	0.35	17.4%	13.0%	0.17
Smoking (%)	28.5%	29.0%	26.7%	0.74	29.2%	30.1%	0.84
CCI, mean (range)	5.4 (4.0–6.0)	5.4 (4.0–6.0)	5.3 (5.0–6.0)	0.81	5.4 (4.0–6.0)	5.4 (5.0–6.0)	0.93
Line of pembrolizumab (%)							
1	63.6%	63.4%	64.3%	0.90	63.6%	58.3%	0.22
≥2	36.4%	36.6%	35.7%		36.4%	41.7%	
Concomitant chemotherapy (%)	54.5%	50.0%	69.6%	<0.01	54.2%	50.6%	0.43
ECOG (%)							
<2	86.8%	86.0%	89.3%	0.52	86.%	88.5%	0.39
≥2	13.2%	14.0%	10.7%		14.0%	11.5%	
Stage (%)							

3	13.2%	11.8%	17.9%	0.24	11.4%	13.7%	0.44
4	86.8%	88.2%	82.1%		88.6%	86.3%	
Histologic features (%)							
Adenocarcinoma	67.7%	67.7%	67.9%	0.92	68.8%	76.0%	0.19
Squamous	24.4%	4.7%%	23.2%		23.6%	18.5%	
Others	7.9%	7.6%	8.9%		7.6%	5.5%	
Metastasis status (%)							
Brain	14.9%	15.6%	12.5%	0.56	15.6%	15.8%	0.93
Bone	31.0%	32.3%	26.8%	0.43	33.1%	34.1%	0.79
Liver	12.0%	10.7%	16.0%	0.28	13.1%	8.9%	0.13
Others	84.7%	84.9%	83.9%	0.85	86.3%	86.3%	0.99
PD–L1 tumor proportion score (%)							
<1%	11.6%	11.3%	12.5%	0.11	11.7%	9.5%	0.60
1–49%	21.9%	18.8%	32.2%		20.7%	22.5%	
≥50%	47.1%	50.5%	35.7%		47.9%	51.6%	
Unknown	19.4%	19.4%	19.6%		19.7%	16.4%	
Comorbidity							
Hypertension (%)	28.5%	30.1%	23.2%	0.31	28.9%	36.4%	0.06
Diabetes (%)	14.8%	16.1%	10.7%	0.31	14.6%	15.7%	0.75
Dyslipidemia (%)	11.9%	10.7%	16.1%	0.28	12.4%	8.5%	0.14
Ischemic heart disease (%)	5.3%	4.8%	7.1%	0.50	5.2%	2.4%	0.09
Heart failure (%)	1.6%	2.1%	0.0%	0.26	1.8%	0.0%	0.07
Cerebrovascular disease (%)	4.5%	3.8%	7.1%	0.28	4.5%	2.7%	0.27
Hypothyroidism (%)	0.4%	0.5%	0.0%	0.58	0.4%	0.0%	0.25
COPD (%)	21.4%	24.7%	10.7%	0.02	22.4%	22.8%	0.90
Chronic kidney disease (%)	7.0%	7.5%	5.4%	0.57	8.1%	12.5%	0.11
Biochemical data							
eGFR (mL/min/1.73 m^2)	98.7 (71.6–118.2)	99.3 (71.2–117.6)	96.6 (75.1–119.1)	0.68	100.7 (71.2–118.2)	87.2 (67.9–111.7)	0.06
ALT (U/L)	29.0(14–37)	28.6 (14.0–36.0)	30.6 (17.0–41.0)	0.62	28.7 (14.0–36.0)	30.1 (18.0–41.0)	0.63
AST (U/L)	31.4(19–33)	31.6 (19.0–33.0)	30.7 (19.0–36.0)	0.86	32.0 (19.0–33.0)	28.4 (19.0–36.0)	0.29
Total bilirubin (mg/dL)	0.6 (0.4–0.6)	0.6 (0.4–0.6)	0.6 (0.4–0.7)	0.68	0.6 (0.4–0.6)	0.6 (0.4–0.7)	0.94
Fasting glucose (mg/dL)	126.0(97–132)	128.7 (97.0–134.0)	116.3 (96.0–123.0)	0.22	124.5 (97.0–132.0)	118.0 (99.0–121.0)	0.38
HbA1c (%)	6.2 (5.6–6.5)	6.2 (5.6–6.5)	6.1 (5.6–6.4)	0.69	6.2 (5.6–6.5)	6.2 (6.0–6.6)	0.88
WBC (10^3/uL)	8.8 (5.9–10.9)	9.1 (5.9–11.1)	7.9 (5.8–9.6)	0.02	9.1 (5.8–11.1)	8.5 (6.4–10.2)	0.29
Hemoglobin (g/dL)	11.6 (10.5–13.0)	11.6 (10.5–13.2)	11.5 (10.6–12.7)	0.67	11.6 (10.5–13.2)	11.6 (10.6–12.6)	0.76
Platelet (10^3/uL)	273.7 (187.5–328.5)	275.0 (194.5–327.5)	269.4 (178.5–344.0)	0.77	271.3 (186.0–323.0)	269.9 (191.0–344.0)	0.92

Abbreviations - CCI: Charlson comorbidity index, ECOG: Eastern Cooperative Oncology Group, PD-L1: programmed death ligand 1, COPD: chronic obstructive pulmonary disease, eGFR: estimated glomerular filtration rate, ALT: alanine aminotransferase, AST: aspartate aminotransferase, TSH: thyroid-stimulating hormone, WBC: white blood cell. Note: Continuous variables are expressed as mean (Q1-Q3) and dichotomous variables are expressed as percentage (%) *Since the patients were adjusted by IPTW, the total patient number was not identical to that in the original cohort.

Table S5. Summary of immune-related adverse events.

IrAEscategory	Total Patients (*n* = 242)	Standard-Dose (*n* = 147)	Low-Dose (*n* = 95)	*p* Value
AllirAEs	148 (61.1%)	94 (63.69%)	54 (56.8%)	0.26
SkinirAEs	72 (29.7%)	46 (31.2%)	26 (27.3%)	0.51
HepaticirAEs	66 (27.2%)	45 (30.6%)	21 (22.1%)	0.14
DiabeticirAEs	28 (11.5%)	14 (9.5%)	14 (14.7%)	0.21
ThyroidirAEs	39 (16.1%)	23 (15.6%)	16 (16.8%)	0.80
Seriousadverseevents*	70 (28.9%)	42 (28.5%)	28 (29.4%)	0.87

Abbreviation: irAEs: immune-related adverse effects *Receive intravenous steroid uses more than 3 days.

Table S6. Baseline characteristics of our study and Low JL et al. study between fixed-dose 200mg and 100 mg group.

	Our Study			Low JL et al.[1]		
	Fixed–Dose 200 mg (*n* = 47)	Fixed–Dose 100 mg (*n* = 107)	*p* Value	Fixed–Dose 200 mg (*n* = 49)	Fixed–Dose 100 mg (*n* = 65)	*p* Value
Age, median years (range)	62.0 (57.0–73.0)	62.0 (55.0–73.0)	0.25	60.5 (28.4–80.0)	69.9 (42.8–92.2)	<.001
Male (%)	74%	48%	<0.001	84%	69%	0.084
Body weight, median kg (range)	68.0 (59.3–75.0)	53.2 (48.2–61.7)	<0.001	59 (37–103)	59 (31–101)	0.245
Smoking (%)	38%	16%	<0.001	65%	75%	0.298
Line of pembrolizumab (%)						
1	60%	64%	0.639	88%	74%	0.223
≥2	40%	36%		12%	26%	
Concomitant chemotherapy (%)	23%	60%	<0.001	65%	26%	<0.001
ECOG (%)						
< 2	85%	88%	0.640	86%	71%	0.053
≥2	15%	12%		14%	29%	
Histologic features (%)						
Adenocarcinoma	60%	71%	0.313	76%	68%	0.553
Squamous	10%	6%		14%	9%	
Others	30%	23%		10%	23%	
PD–L1 tumor proportion score (%)						
<1%	9%	14%	0.063	22%	8%	0.005
1–49%	13%	21%		37%	20%	
≥50%	68%	43%		39%	68%	
Unknown	10%	22%		2%	4%	
Dose/kg of pembrolizumab, median (range)	2.9 (2.66–3.37)	1.87 (1.62–2.07)	<0.001	2.87 (1.94–4.98)	1.85 (1.24–3.20)	<0.001

Abbreviations - ECOG: Eastern Cooperative Oncology Group, PD-L1: programmed death ligand 1.

Reference

1. Low, J.L.; Huang, Y.; Sooi, K.; Ang, Y.; Chan, Z.Y.; Spencer, K.; Jeyasekharan, A.D.; Sundar, R.; Goh, B.C.; Soo, R.; et al. Low-dose pembrolizumab in the treatment of advanced non-small cell lung cancer. *Int J Cancer* **2021**, doi:10.1002/ijc.33534.
